# Congruence and trajectories of device-measured and self-reported physical activity during therapy for early breast cancer

**DOI:** 10.1007/s10549-021-06195-7

**Published:** 2021-03-31

**Authors:** H. Helbrich, M. Braun, C. Hanusch, G. Mueller, H. Falk, R. Flondor, N. Harbeck, K. Hermelink, R. Wuerstlein, S. Keim, F. Neufeld, S. Steins-Loeber, K. Haertl

**Affiliations:** 1 Psychology School, Hochschule Fresenius, University of Applied Sciences, Infanteriestraße 11a, 80797 Munich, Germany; 2 Department of Gynecology, Breast Center, Rotkreuzklinikum, Munich, Germany; 3 Breast Center, Department of Gynecology and Obstetrics and CCCMunich, LMU University Hospital, Munich, Germany; 4 Breast Center, Department of Gynecology and Obstetrics, Helios Klinikum Munchen West, Munich, Germany; 5 Department of Clinical Psychology and Psychotherapy, University of Bamberg, Bamberg, Germany

**Keywords:** Breast cancer, Physical activity, Primary treatment, Accelerometer, Activity monitoring, Self-report

## Abstract

**Purpose:**

This study examines congruence between self-reported and device-measured physical activity data in women with early breast cancer and compares trajectories under different treatments.

**Methods:**

Women with non-metastatic breast cancer were recruited before primary therapy. In four weeks distributed over six months after treatment start, patients reported time spent on work, transport, chores and sports via diary and wore Garmin^®^ vivofit 3 accelerometers to assess steps taken. Associations between these measures and agreement regarding guideline adherence were tested with Spearman’s Correlation Coefficient and Weighted Kappa statistic. Effects of time and treatment were evaluated using mixed analyses of variance.

**Results:**

Ninety-nine participants (median age *=* 50) were treated with adjuvant (*N* *=* 23), neoadjuvant (*N* *=* 21) or without chemotherapy (*N* *=* 55). Coherence between self-report and device data was strong (r *=* 0.566). Agreement about reaching recommendations was only “fair” (kappa coefficient *=* 0.321 and 0.249, resp.). Neither treatment or week nor their interaction had effects on step counts (all *p* > 0.05). Self-reported activity time was lower for patients with chemotherapy than for those without (adjuvant: ∆ *=* 69min, *p* *=* 0.006, neoadjuvant: ∆ *=* 45min, *p* *=* 0.038) and lower in week 18 than in week 3 (∆ *=* 43min, *p* *=* 0.010).

**Conclusion:**

Results show that consumer-grade activity monitors and self-reports correlate but show different perspectives on physical activity in breast cancer patients. In general, patients perceive some decline regardless of primary treatment regimen. Those affected should be offered assistance to gain the benefits of activity. Accelerometers may help professionals to identify these individuals and patients to verify appraisal of their activity levels.

**Supplementary Information:**

The online version contains supplementary material available at 10.1007/s10549-021-06195-7.

## Introduction

In 2018, approximately 400,000 women in Europe received a breast cancer diagnosis [1]. Disease and treatment result in troubling concomitants and long-term effects [2–5]. Physical activity (PA) is linked to benefits regarding physical and psychological health [3–7] and is recommended from diagnosis. Some guidelines refer to a time frame, e.g. the American College of Sports Medicine (ACSM) and others [8, 9] state 150 min of PA per week. Others use steps to quantify PA and adopt the popular recommendation of 10000 steps daily to patients [10] or transferred the ACSM suggestions into 6286 steps [11]. However, many patients do not meet these recommendations [4, 11–13], so further research is required to enhance understanding of the mechanisms underlying this discrepancy.

PA is often assessed retrospectively by questionnaires retrieving up to several years [14]. They are highly accepted and economical but can be biased due to social desirability, memory distortion and incompleteness of activities assessed [14–17]. Continuous sampling of PA in real time compensates for these limitations. In free-living conditions, pedometers, accelerometers and activity monitors are used for this purpose [14, 18]. Different techniques have been compared in various populations [14, 17–19]. Most studies use correlations between different units to show criterion validity [14]. More infrequently, data from several assessments have been converted into the same unit, resulting in time, steps and energy expenditure being converted into each other [14, 20–25]. Generally, good agreement between methods is found [14] and over- [25] or underestimation [20] are not systematic. Energy expenditure is an often used denominator as it easily calculated from time spent with an activity [26], but deriving it from step counts is prone to errors [27]. Steps and time have been examined less and standard conversion procedures are missing, but they would more understandable for patients than the abstract units of energy expenditure when deriving activity recommendations [28].

PA questionnaires are also common with cancer patients [3, 4, 6, 12, 13, 29–33]. Combination with activity trackers show patients seem more inclined towards overestimation [34–36] than underestimation [37]. Importantly, while in some studies results were independent of the mode of PA assessment [37, 38], others found differences for one but not for the other: In a study by Goedendorp et al. [39], groups differed in their self-reported PA but not when compared via Actometer. Inversely, Rogers et al. [7] found an intervention effect on accelerometer data but not on questionnaires. These differences have received too little attention.

Observational studies on PA in breast cancer mostly focus on changes from pre- to post-treatment. Usually they find that PA decreases [3, 4, 12, 13, 29, 30, 40] and is lower than in non-cancer controls [13, 41]. While the decline may be temporary [12, 29, 33], women frequently report breast cancer specific obstacles to being active, such as side effects [2] (e.g. fatigue, pain, nausea, lymphedema), which vary in their incidence under different treatments [5, 32]. Accordingly, PA is typically lower during treatment than before and afterwards [4, 12, 13, 29, 32] and differs between regimens [12, 32]. Usually, therapy is assessed as one entity [4, 6, 8, 9, 13], so information about PA trajectories across therapy stages is sparse. Two-times self-report assessment within six months post-surgery suggest an increase of PA [3, 30]. Two detailed studies throughout chemotherapy showed a decline during the first half that levelled off [42] or rebounded [31] towards the end of treatment. To our knowledge, there is only one study that assessed PA with devices for more than two weeks [42] and no comparable research on other therapy regimens.

Thus, in a prospective longitudinal repeated measures design, we assessed PA in high resolution with questionnaires and accelerometers repeatedly within 6 months of primary treatment. We investigate whether subjective and device-measured PA correspond during primary therapy of breast cancer and compare trajectories of spontaneous PA in patients without chemotherapy and those with neoadjuvant or adjuvant chemotherapy.

## Methods

### Patients

Eligibility criteria were being female and aged 18 to 70 years, ability to read documents in German, reporting no major medical or psychiatric disorder, having a histologically confirmed primary diagnosis of carcinoma in situ or breast cancer without metastasis and not having started systemic therapy (chemotherapy, antihormonal/antibody therapy) or radiotherapy. According to the research question, patients were assigned to one of three groups: no chemotherapy (NC), adjuvant (AC) or neoadjuvant chemotherapy (NAC) group.

During recruitment between April 2017 and March 2019, breast care nurses, physicians and psychooncologists invited eligible women who attended appointments in one of four participating hospitals personally and via print materials. Interested individuals were offered an in-person briefing where they received comprehensive information and signed informed consent.

### Data collection

This study was performed according to the principles of the Declaration of Helsinki. Study design was approved by the ethics committees of the Ludwig Maximilian University of Munich (Date: 2017/07/05. No:17-308) and of the Hochschule Fresenius, University of Applied Science.

During briefing, participants received an accelerometer and the baseline questionnaire. Two weeks after initial chemotherapy treatment or, if not applicable, four weeks post-surgery, patients started the first week of activity assessment by diary and accelerometer. Questionnaires were sent in advance and returned in self-addressed envelopes. This procedure was repeated 8, 14 and 20 weeks later.

Baseline assessment included demographics and lifestyle before illness. Cancer and treatment data were obtained through hospital reports. Existing daily PA self-reports lacked differentiation between activities or reporting activities less than 15 min [43, 44]. Thus, we developed a diary based on the International Physical Activity Questionnaire [45], referring to one day and adjusting the requested activities—details on development and pretesting with 23 subjects are described in Supplementary Text 1 and Supplementary Table 3. Device-measured activity data were obtained via Garmin^®^ vivofit 3 wristband (Garmin Ltd., Schaffhausen, Switzerland), a commercially available fitness tracker. It is worn continuously day and night on the non-dominant wrist and registers steps via accelerometry. Patients transferred data via the Garmin application using their smartphones, or, if that was not possible or desired, study staff scheduled a meeting for transfer.

### Data analyses

Datasets generated and analyzed during the study are available from the corresponding author on reasonable request. Data analyses were conducted using Microsoft Excel 2016 and SPSS 26. Patient data were analyzed if at least the baseline questionnaire and two of four weeks of activity tracking had been completed.

Self-reported activity time was added up across activities that yielded step counts (walking, running, hiking, step aerobics, dancing, stairclimbing, etc.). To calculate steps from self-reports, we used the metabolic equivalent (MET) rate assigned [26] to each activity yielding steps: based on approximate correlation between speed and METs (i.e. running at 8 km/h yields 8METs, running at 11km/h yields 11METs), 1 MET was equated to 28 steps/min which approximates the conversion ratio of 31steps/min found by Marshall et al. [28]. Steps were calculated per activity as: minutes × MET-rate × 28, and then summed up across walking-related activities.

Accelerometer data as exported from the device interface consisted of 96 segments á 15 min for each day. These were summed up to a daily score if no more than 8 segments were missing and the sum of steps was > 500. Time in segments > 1000 steps was calculated per day for device-measured estimation of minutes spent on intense walking. Weekly averages of daily variables were calculated if no more than one daily score was missing.

Except for mixed analysis of variance, nonparametric tests were used due to violation of parametric assumptions. Statistical tests were performed two-sided at the 5% and 1% level, divided by the number of tests per question (four for comparison of the two measures, two for influence of time and treatment) as adjustment for multiple testing.

Descriptive statistics (frequency, median, interquartile range (IQR), mean, standard deviation) were used to summarize data. Kruskal-Wallis-tests (with Mann-Whitney posthoc-tests) and χ^2^-tests were carried out to check for differences between treatment groups regarding sociodemographic and health characteristics.

Coherence between accelerometer and questionnaire data was analyzed on single day level with data from treatment groups combined by calculating Spearman’s Correlation Coefficient, interpreted as moderate (0.30–0.49) or strong (≥0.50). We tested concordance regarding the number of patients who reached the recommended thresholds of 6286 [11] and 10000 [10] steps and the equivalent [28] of 62 and 100 min of walking activity, using Cohen’s Kappa (κ). Relevant interpretation thresholds were: slight agreement (0.01–0.20), fair agreement (0.21–0.40) and moderate agreement (0.41–0.60) [46].

To analyze trajectories of device-assessed and self-reported PA in patients with different treatments, mixed analyses of variance with treatment group as between- and week as within-subject factor were calculated, with step count and activity minutes as respective dependent variables, and Tukey-HSD posthoc-tests.

## Results

Of 112 patients enrolled, 12 dropped out before the activity-monitoring phase and one after the first week. In total, the remaining 99 patients handed in 375 of 396 (95%) weekly protocols. Accelerometer step counts were transferred for 2412 of 2772 days (87%). No participant reported unplanned hospitalization due to complications during study participation, but 12 NC patients had inpatient stays at rehabilitation centres. Radiation took place in 38 of the weeks reported.

Baseline sample characteristics are shown in Table 1. Most sociodemographic and general health characteristics were balanced between treatment groups, while tumour-related variables differed.

**Table 1:** Sociodemographic and health-related patient characteristics

		Total (*n* = 99)	NC (*n* = 55)	AC (*n* = 23)	NAC (*n* = 21)	*p*
Age, years	median	50	53	48	49	0.051^a^
	(IQR)	(45–56)	(47–57)	(43–56)	(40–55)	
Marital Status, n(%)	partner	72 (73)	39 (71)	16 (70)	17 (81)	0.623^b^
Children, n(%)	yes	75 (76)	43 (78)	18 (78)	14 (67)	0.594^b^
Education, n(%)	≥ 13 years	59 (60)	31 (56)	12 (52)	16 (76)	0.215^b^
Working prior to diagnosis, n(%)	yes	77 (78)	41 (75)	16 (70)	20 (95)	0.094^b^
Economic situation (self-reported), n(%)	very good	22 (22)	8 (15)	6 (26)	8 (38)	0.040^ad^
	good	59 (60)	34 (62)	13 (57)	12 (57)	
	ok	9 (9)	8 (15)	1 (4)	0	
	precarious	9 (9)	5 (9)	3 (13)	1 (5)	
Menopausal status, n(%)	pre	38 (38)	20 (39)	7 (39)	11 (52)	0.600^b^
	peri	14 (14)	7 (14)	4 (22)	3 (14)	
	post	38 (38)	25 (48)	7 (39)	6 (29)	
BMI, kg/m^2^	median	23	23	24	21	0.213^a^
	(IQR)	(21–26)	(21–27)	(21–26)	(20–26)	
Time since diagnosis, days	median	52	56	67	36	< 0.001^ade^
	(IQR)	(36–73)	(42–73)	(52–87)	(28–46)	
UICC tumour stadium, n(%)	0	11 (11)	11 (20)	0	0	< 0.001^acd^
	I	49 (51)	32 (58)	7 (32)	10 (50)	
	II	32 (33)	11 (20)	12 (59)	8 (40)	
	III	5 (5)	1 (2)	2 (9)	2 (10)	
Operation received, n(%)	breast-preserving	73 (74)	47 (86)	19 (83)	7 (33)	< 0.001^bde^
	mastectomy	12 (12)	8 (15)	4 (17)	0	
	none	14 (14)	0	0	14 (67)	
Axillary Dissection, n(%)	yes	8 (8)	2 (4)	6 (26)	0	0.002^bce^
Chemotherapy, n(%)	Paclitaxel	30 (30)	–	16 (70)	14 (67)	0.881
	Docetaxel	5 (5)	–	2 (9)	3 (14)	0.537
	Cyclophosphamide	22 (22)	–	13 (57)	9 (43)	0.342
	Epirubicin	26 (26)	–	14 (61)	12 (57)	0.836
	Carboplatin	10 (10)	–	0	10 (48)	< 0.001^e^
	Cisplatin	2 (2)	–	1 (4)	1 (5)	0.935
Radiation, n(%)	yes	56 (57)	42 (76)	8 (35)	6 (28)	< 0.001^bcd^
Antihormonal therapy, n(%)	yes	43 (43)	37 (67)	3 (13)	3 (14)	< 0.001^bcd^
Antibody therapy, n(%)	yes	13 (13)	1 (2)	3 (13)	9 (43)	< 0.001^bde^

*NC *no chemotherapy, *AC adjuvant chemotherapy*, *NAC* neoadjuvant chemotherapy, *IQR* interquartile range, *BMI* body mass index, *UICC* Union for International Cancer Control

^a^Kruskal–Wallis-Test with Mann–Whitney posthoc-tests

^b^χ^2^-Test

^c^Significant difference between NC and AC

^d^Significant difference between NA and NAC

^e^Significant difference between AC and NAC

Across all treatment groups and days, median accelerometer step count was 8765 with IQR [5905, 12183] and accelerometer-based estimation for time spend highly active was 15 min with IQR [0, 45]. Patients self-reported spending about one hour (median *=* 60, IQR [10, 105]) per day with activities increasing step count which was calculated to correspond to 8904 (IQR [1904, 17136]) steps. Coherence between steps counted by accelerometer and diary-reported minutes spent on walking type activities was r *=*0.566 (p < 0.0001). Agreement about reaching recommended thresholds for steps was fair (κ=0.321; p < 0.0001). Classification conformed in 55% (1281) of cases, 19% (441) of classifications according to diary data exceeded the device’s step count and 26% (600) underestimated it. Agreement about reaching recommended thresholds for walking time was fair (κ *=* 0.249; p < 0.0001). Classification conformed in 66% (1514) of cases, 32% (735) of classifications according to diary data exceeded accelerometer-assessed walking time and 2% (48) underestimated it.

**Fig. 1:**
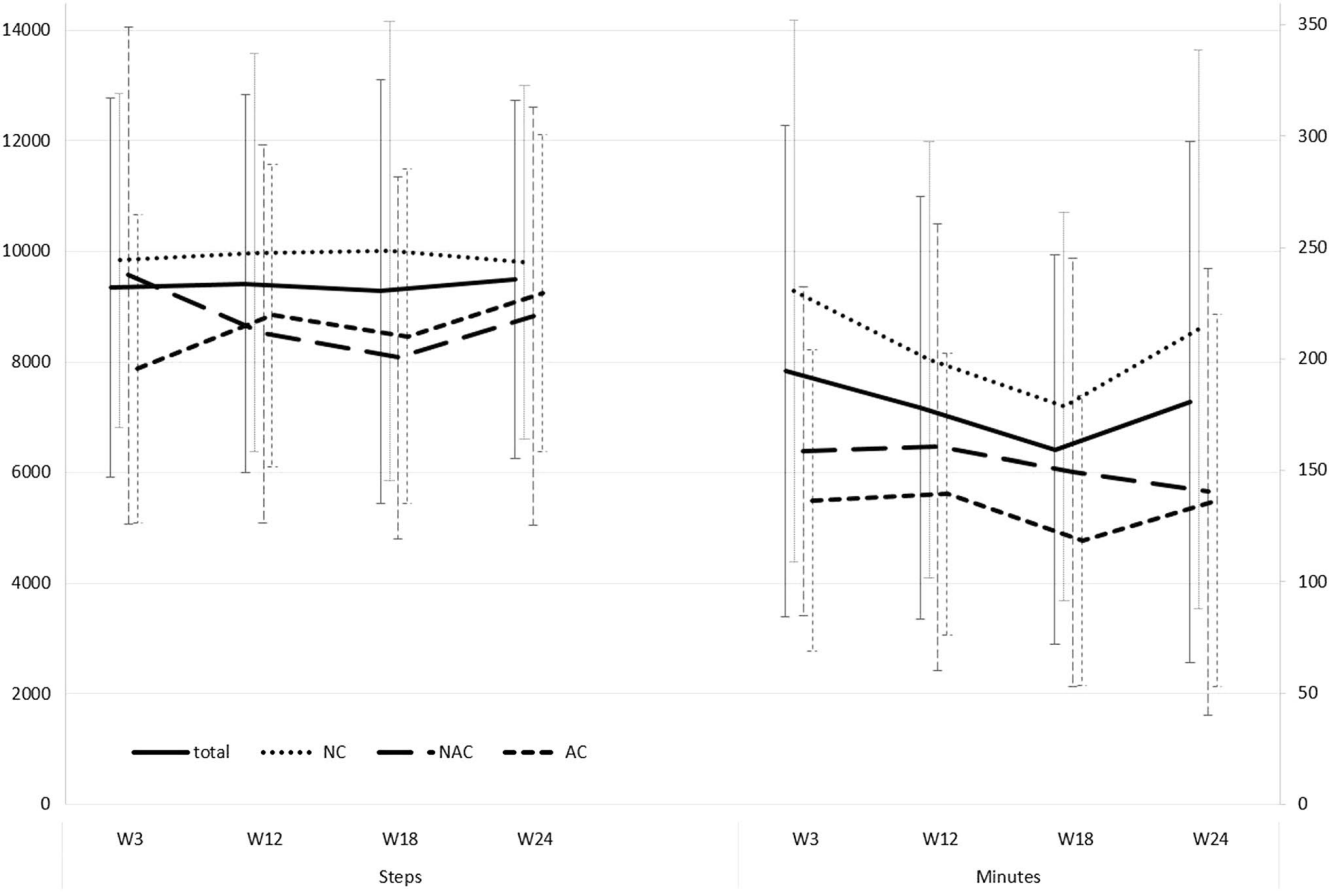
Recorded steps and self-reported minutes of physical activity in week 3, 12, 18 and 24 of primary breast cancer treatment. *Total* = all patients, *n* = 99; *NC* no chemotherapy,* n* = 55; *AC* adjuvant chemotherapy,* n* = 23; *NAC* neoadjuvant chemotherapy,* n* = 21

Average daily step counts (Fig. 1; Supplementary Table 4) ranged from 7877 for AC in week 3 to 10015 for NC in week 18. Variance was high with standard deviations ranging from 2734 to 4494. Inferential statistics (Table 2) did not show systematic effects of either treatment group or time nor their interaction on step counts.

**Table 2:** Effects of treatment group as between- and week as within-subject-factor on accelerometer recorded steps and self-reported minutes of physical activity during primary breast cancer treatment (mixed analyses of variance)

	Steps			Minutes		
	*df*	*F*	*p*	*df*	*F*	*p*
Between subjects effects						
Treatment	2	0.636	0.533	2	6.475	0.003
Error (treatment)	61			66		
Within subjects effects						
Time	3	1.309	0.273	3	3.239	0.023
Time*treatment	6	0.876	0.514	6	1.122	0.351
Error (time)	183			198		

*Treatment* adjuvant, neoadjuvant or no chemotherapy, *Time* week 3, 12, 18 and 24 of primary treatment

Average self-reported PA time per day (Fig. 1; Supplementary Table 4) ranged from 118 min for AC in week 18 to 231 min for NC in week 3. Variance was high with standard deviations ranging from 63 to 125 min. Inferential statistics showed systematic effects of treatment group and time but not their interaction on self-reported PA minutes (Table 2). NC reported more activity minutes than AC (∆ = 69 min, *p* = 0.006, CI [19, 119]) and NAC (∆ = 45 min, *p* = 0.038, CI [4, 93]) which did not differ. Patients reported more activity minutes in week 3 than in week 18 (∆ = 43min, *p* = 0.010, CI [7, 78]), other comparisons between weeks did not show significant differences.

## Discussion

In the first assessment of PA during the first months after treatment start for breast cancer with both a device and a diary, findings provide evidence for fair concordance between both sources. Accelerometer counted steps had large interindividual differences with no systematic influence from time since start or type of treatment, while self-reported PA first declined, then rebounded and was lower under chemotherapy.

Correlation between device-based and subjective assessments was “strong” and thereby higher than in other studies on breast cancer [36, 47]. Agreement about adherence to both activity recommendations classified as “fair”: a narrow majority of self-reports conformed to device data ratings. Discrepancies concerning walking time resulted from higher estimates in self-reports. This may be caused by the device’s low resolution of 15 min-segments, but also by patients overreporting PA time [35, 36]. For step recommendation adherence, higher self-report- and device-based ratings were equally common. The latter may result from people underreporting light activity and steps taken [17, 37]. In addition, no established procedure for converting activity time into steps was available, and though we calculated a similar conversion ratio as others [29], our method may be imprecise. Confirming the reasons for deviations would require a gold standard PA measure, but fair overall agreement already offers insights into the transferability of information between questionnaires and accelerometers.

Step counts were high with a median of 8765 per day, surpassing numbers previously reported during breast cancer therapy [6, 48], even within walking interventions [11, 49]. Self-reported PA was high as well, with a median of 150 min per day. While many studies only assessed exercise [12, 29, 40], those covering more PA domains still reported less than 1h of PA (or equivalent energy expenditure) per day [3, 4, 13, 50]. It is noteworthy that some studies using more elaborate questionnaires found higher activity levels [6, 30, 32], e.g. 70 min per day [31]. As device-based assessments confirm the high level of PA in our patients, these findings do not merely result from overreporting. Together with studies showing 50–80% adherence to PA guidelines [50, 51] even during chemotherapy [31], they suggest that high levels of PA are possible even under straining treatment.

Large interindividual differences in PA that were observed in our study have been reported previously among breast cancer patients [4, 13, 29]. These differences may contribute to the fact that analyses of systematic differences in PA were inconclusive. It was surprising that self-reported PA but not step counts differed between treatment groups and assessment time.

NC patients reported more active time than patients under chemotherapy while their step counts did not differ. Most studies have found less self-reported PA for chemotherapy patients [4, 12, 13, 32], though there are findings that treatment makes no difference for leisure-time exercise [29]. The present results suggest that patients under chemotherapy perceive a greater decline of their PA than those without chemotherapy which is not objectively confirmable. No previous research used device-based data for comparison or compared AC and NAC. Though NAC patients had not had surgery yet, their subjective and device-measured activity level was comparable to AC patients.

Patients reported less PA time in week 18 than in week 3 while, again, step counts showed no difference. While patient-reported PA has often been shown to recede from pre- to post-treatment [12, 30, 40], this is the first prospective observation of this decline in NC and NAC patients specifically. In contrast, two studies without discrimination between regimens suggested a rise of self-reported PA throughout the year after surgery [3, 30], but one had 4-month intervals only and included patients with a PA intervention [3]. Studies with device-measured data showed a decline of PA from the beginning of chemotherapy [31, 42]. Our findings suggest that the decrease of PA may not occur suddenly but rather as a process continuing for months after treatment start. Data show that PA increased again in the last interval, which might mark the onset of PA rehabilitation reported previously [4, 12, 29, 31, 40], although later than others have suggested [3, 30].

Contrary to expectations, treatment groups did not differ significantly in their trajectories of PA during therapy in either measure even though differences are reported in PA after [12, 32] and in distress during therapy [5, 32]. The chart indicates there may be differences that were concealed by high interindividual variance. It also suggests that, while device-measured and self-report PA intercorrelated, they had different trajectories. To our knowledge, no previous study has compared changes between both assessments. As they target different aspects of PA, it is possible that, while the time patients spend physically active changes, step count may remain the same (or reverse) if the type of PA or other lifestyle factors change.

When interpreting these results, there are some limitations to be considered. While Cohen’s kappa provides information about absolute agreement, other statistics have also been recommended for activity monitor validation [52]. While we could not use these as many patients reported no activities at all that yielded steps, they would be useful for subsequent studies.

Study participants mostly described themselves as well situated and of healthy weight, which is not representative for all breast cancer patients [5, 12, 33]. In general, patients with high levels of functioning rather are approached by recruiting staff and agree to participate in clinical studies [53]. Co-operation partners encouraged participation in all patients equally, but as participation was voluntary, a selection bias may have occurred. Education, social support and lower weight are characteristics linked to more PA [3, 6, 11, 33], which is reflected in the high numbers of steps and self-reported activity minutes in our sample. Although this may limit the generalizability of the findings, especially about PA trajectories, it does not invalidate results for this sample. For future studies with larger sample sizes, it would be interesting to also analyze effects of different chemo- and radiotherapy regimen and other interindividual differences like rehabilitation that all affect activity levels.

Some potential problems can be seen regarding the accelerometer. Consumer-grade activity monitors have limited accuracy when compared to research-grade devices [10, 54], so step data might be distorted. Still, among consumer-grade monitors, Garmin^®^ Vivofit 3 showed comparatively high precision [55, 56]. As discrepancies between device-based and self-report assessments occurred in a study with research-grade devices [57] as well, our findings confirm that even commercially available accelerometers provide additional information to questionnaires. It is to be noted that the 15-min-interval temporal resolution of the Garmin^®^ Vivofit 3 may be too low for some research questions. Otherwise, the device proved to be a useful tool with good acceptance and adherence with no participants reporting adverse effects and data transfer resembling that of studies with other consumer-grade monitors [11].

Study materials emphasized that participation should not be a reason for patients to change their activity level. Influence of social desirability was avoided by postal delivery and pseudonymization of questionnaires. Yet, for some patients, study involvement may have been stimulating to be more active. Receiving information or answering questionnaires about PA and wearing an accelerometer can increase motivation and actual activity in cancer patients [31, 38]. Hence, study participation would have served as an intervention itself. This is likely as some patients declared interest in purchasing their own activity monitor. Independent of study involvement, activity may also have been promoted by professionals, or by the illness being a motivator for lifestyle changes. These factors may have contributed to the high PA levels observed.

For research on PA in breast cancer, accelerometers are recommendable. As they spare patients the need for recall and protocolling, they facilitate continuous monitoring for long periods. Deviations between device-based assessment and self-report must be considered when interpreting research findings. It is important to note that both assessments carry valid information. Perceived PA may be more important when researching psychological factors such as self-efficacy, while the device-measured amount of PA is preferential when analyzing physical aspects. The nature of the difference itself should also be examined, e.g. to improve accuracy of self-reports and to understand what role the perception of one’s PA plays when self-efficacy and coping with cancer are discussed. Better agreement with device-measured data for our questionnaire compared to a common 4-item questionnaire [58] implies that more elaborate surveys can enhance self-report accuracy. As for trajectories of PA, future studies with larger samples and continuous assessment should specify how long the decline continues after the start of treatment and when recovery begins. Linking these changes to external circumstances may help to identify causes and tailor appropriate interventions to enhance PA and/or avoid its decline.

Results imply that practitioners can use both questionnaire and accelerometer data to estimate whether breast cancer patients meet activity recommendations, address the topic and make suggestions about change. Using steps as a unit in communication has advantages as they are less abstract that energy expenditure and easier to log with consumer-grade trackers that activity time, which the Garmin^®^ Vivofit 3 may measure less precisely. Clinicians should also consider differences between device-measured and self-report PA data. High average PA levels with large interindividual differences prove that it is generally possible to stay active during primary treatment, a piece of information that may motivate patients to do so, but individual assessment and recommendations are necessary. Interventions should consider that the PA decline is an ongoing process throughout primary therapy that may be slowed down or avoided, instead of a rapid decay after diagnosis that has to be reversed. Participants’ interest in the accelerometer matches findings in intervention studies [38, 49], showing they are a useful tool to promote PA. As NAC patients, like AC patients, report lower PA under chemotherapy, both may need support for PA maintenance [2].

To our knowledge, this is the first prospective study to repeatedly assess PA both via devices and self-report during the first six months of primary treatment for early breast cancer. Our findings on concordance between assessments and on activity patterns may help when interpreting results in PA research and tailoring interventions to support PA in breast cancer.

## Supplementary Information

Below is the link to the electronic supplementary material.

Supplementary file1 (PDF 150 kb)

## Data Availability

Datasets generated and analyzed during the study are available from the corresponding author on reasonable request.
