# Supplementary material for: Congruence and trajectories of device-measured and self-reported physical activity during therapy for early breast cancer
Source: Breast Cancer Res Treat. 2021 Mar 31;188(2):351–9. doi: 10.1007/s10549-021-06195-7 (PMC8260526; doi:10.1007/s10549-021-06195-7)
Supplement: Supplementary file 1 — Supplementary file1 (PDF 150 kb) [file 10549_2021_6195_MOESM1_ESM.pdf]

Helbrich H, Braun M, Hanusch C et al (2021) Congruence and trajectories of device-measured and self-reported physical activity during therapy for early breast cancer. Breast Cancer Res Treat. DOI: 10.1007/s10549-021-06195-7.

Supplementary text

Supplementary Text 1: Validation of the activity diary

This text describes how the physical activity diary was developed and tested. Existing daily PA self-reports lacked differentiation between activities or reporting activities less than 15min [1, 2]. Thus, we developed a diary based on the International Physical Activity Questionnaire (IPAQ) [3] and the Global Physical Activity Questionnaire (GPAQ) [4]. Both underlying questionnaires have a similar structure and refer to the previous or a typical week. Different activities are explained and illustrated by examples. The IPAQ names physical activity for work (moderate and vigorous), traveling by motor vehicle/by bike/by foot, gardening (moderate and vigorous), chores, going for walks, sports (moderate and vigorous) and sitting, the GPAQ only refers to a subset of those domains. In both, the subject is asked whether they engage in said activity and if they do, they are asked how many times a week and how long on average they spent with said activity, which is to be answered in hours and minutes.

To determine relevant areas of physical activity, we checked that both questionnaires concordantly address hard and moderate working activity, walking and biking for transport, and sports. We adopted these domains and their explanations as well as chores, gardening and going for walks from the more elaborate IPAQ but skipped themes like sitting and motorized transport which were not of interest to us. For gardening, we omitted the differentiation between moderate and vigorous activities. For sports, instead of discriminating moderate and vigorous intensity, participants were asked to specifically name all exercises they had done (giving both the examples for moderate and vigorous leisure PA from the IPAQ and GPAQ) in the spaces provided. As the diary is completed daily, we also omitted the question on how many days the person carried out each activity, but moved straight on to the question how much time (in hours and minutes) they spent with the activity that day.

The IPAQ [3, 5–8] and GPAQ [4, 9–11] have been extensively validated in different populations, so with the minor changes made there is no reason to expect less validity or reliability for the modified diary version. In contrast, while the IPAQ requires to classify gardening or sports activities into moderate or vigorous PA, the diary did not require this differentiation from the subject – it was skipped for gardening and for the sports domain, by asking subjects to just name their sports activities, transferred to the study staff. As any difficulties subjects might have with the classification do not apply, validity would rather be expected to improve. As the GPAQ has satisfactory psychometric properties without the activities omitted from the IPAQ, the omission should not disturb assessment of the other pastimes. Additionally, the questions about duration of each activity can be answered better for the previous day than for a whole week, as a smaller time lag between the activity and the questionnaire reduces recall errors and patients do no need to calculate averages of durations. Still, we conducted a pretest of the resulting activity diary that consisted of two stages. Comprehensibility was assessed by qualitative feedback from subjects about difficulties caused by ambiguity, wording or other causes.

The first stage was a sample of two breast cancer patients recruited by author Haertl in her practice for ambulatory psychotherapy who declared their readiness to complete the activity diary for a few days and give oral feedback on mayor difficulties that occurred. Each completed a whole week of reporting, and neither reported any problems, so no changes were made at that stage.

The second stage of pretesting the activity diary in more detail was conducted as part of a bachelor thesis supervised by authors Haertl and Helbrich. The student recruited a sample of 23 healthy adult women aged 40 to 60 years (87% married; 12 with university degree, 5 with vocational training, 6 others) who completed the activity diary for 2 weeks and gave written feedback on the completion process and on single items in the end. To evaluate comprehensibility, all items from the activity diary were reprinted in the feedback questionnaire. Participants could tag all which were hard to understand/ ambiguous worded/ caused other problems, and were encouraged to add explanations of the difficulties in the space provided. There was also room for further suggestions or comments.

Congruence and trajectories of device-measured and self-reported physical activity during therapy for early breast cancer. DOI: 10.1007/s10549-021-06195-7.

Of the 23 participants, 21 completed the feedback questionnaire. Supplementary Table 3 shows the number of comments and the comments on each item.

There were few comments altogether and none of the comments reflected a problem that would not occur in the original version of the IPAQ or GPAQ. Thus, as we ranked studies showing adequate psychometric properties in these questionnaires [3–11] more meaningful, we did not make any changes to the diary forms. However, to provide for the problems the subjects mentioned, study staff gave some general advice on the diary orally in the personal invitation appointment. Patients were encouraged to familiarize themselves with the diary before the first assessment to avoid missing the given examples (e.g., childcare can been attributed to “caring for your family”). For each day, they should in advance choose either transport or leisure as a category for which they count each way walked or taken by bike, and they should name all activities that raise heart and breathing rate which indicates moderate or vigorous PA [4, 12, 13] for the sports items.

To further ensure comprehensibility during the study, participants received a call from study staff two or three days into the first week of activity monitoring in which they were asked for problems occurring with the questionnaire. Participants were also encouraged to contact study staff by phone or email if any problems occurred. Few problems were reported and could be settled that way.

In summary, the newly developed diary made only minor changes to the IPAQ/GPAQ and caused few problems in a pretest. Thus, validity and compensability can be assumed.

1. Sallis JF, Haskell WL, Wood PD et al. Physical activity assessment methodology in the Five-City Project. Am J Epidemiol 1985; 121(1):91–106.
2. Wollmerstedt N, Nöth U, Ince A et al. The Daily Activity Questionnaire: A Novel Questionnaire to Assess Patient Activity After Total Hip Arthroplasty. The Journal of Arthroplasty 2010; 25(3):475-480.e3.
3. Craig CL, Marshall AL, Sjöström M et al. International physical activity questionnaire: 12-country reliability and validity. Med Sci Sports Exerc 2003; 35(8):1381–1395.
4. Armstrong T, Bull F. Development of the World Health Organization Global Physical Activity Questionnaire (GPAQ). J Public Health 2006; 14(2):66–70.
5. Vancampfort D, Wyckaert S, Sienaert P et al. Concurrent validity of the international physical activity questionnaire in outpatients with bipolar disorder: Comparison with the Sensewear Armband. Psychiatry Research 2016; 237:122–126.
6. O’Neill B, McDonough SM, Wilson JJ et al. Comparing accelerometer, pedometer and a questionnaire for measuring physical activity in bronchiectasis: a validity and feasibility study? Respir Res 2017. doi:10.1186/s12931-016-0497-2.
7. Hagströmer M, Oja P, Sjöström M. The International Physical Activity Questionnaire (IPAQ): a study of concurrent and construct validity. Public Health Nutr 2006; 9(6):755–762.
8. Vandelanotte C, Bourdeaudhuij ID, Philippaerts R et al. Reliability and Validity of a Computerized and Dutch Version of the International Physical Activity Questionnaire (IPAQ). Journal of Physical Activity and Health 2005; 2(1):63–75.
9. Rivière F, Widad FZ, Speyer E et al. Reliability and validity of the French version of the global physical activity questionnaire. Journal of Sport and Health Science 2018; 7(3):339–345.
10. Keating XD, Zhou K, Liu X et al. Reliability and Concurrent Validity of Global Physical Activity Questionnaire (GPAQ): A Systematic Review. International Journal of Environmental Research and Public Health 2019; 16(21):4128.

Congruence and trajectories of device-measured and self-reported physical activity during therapy for early breast cancer. DOI: 10.1007/s10549-021-06195-7.

11. Hu B, Lin LF, Zhuang MQ et al. Reliability and relative validity of three physical activity questionnaires in Taizhou population of China: the Taizhou Longitudinal Study. Public Health 2015; 129(9):1211–1217.

12. Alqahtani BA, Elnaggar AM, Alhowimel AS, Elnaggar RK. The descriptive pattern of physical activity in Saudi Arabia: analysis of national survey data. Int Health 2020. doi:10.1093/inthealth/ihaa027.

13. Howells K, Wellard I, Woolf-May K. Young children's physical activity levels in primary (elementary) schools: what impact does physical education lessons have for young children? Early Child Development and Care 2020; 190(5):766–777.

Congruence and trajectories of device-measured and self-reported physical activity during therapy for early breast cancer. DOI: 10.1007/s10549-021-06195-7.

Supplementary tables

Table 3. Comments on each item and the general physical activity diary in the pretest, n = 21.

Item	N	Comments
Moderate work	0	-
Vigorous work	0	-
Traveling by bike	1	I only realized that not all biking counts here when I read the sports question which comes later. This is confusing.
Traveling by foot	1	Is “walking to a restaurant” “going from place to place” or “walk in your leisure time”?
Chores	0	-
Gardening	0	-
Going for walks	0	-
Sports (several items)	3	Should I name [specific activity] here? (3x)
General	1	Where should I put childcare?

Note: N = Number of comments received for this item.

Table 4. Recorded steps and self-reported minutes of physical activity in week 3, 12, 18 and 24 of primary breast cancer treatment.

		Week 3	Week 12	Week 18	Week 24
Steps, M (SD)	total	9348 (3423)	9414 (3417)	9275 (3827)	9487 (3245)
	NC	9839 (3027)	9978 (3596)	10015 (4154)	9807 (3204)
	NAC	9567 (4494)	8517 (3419)	8074 (3273)	8826 (3379)
	AC	7877 (2786)	8845 (2734)	8463 (3019)	9244 (2876)
Minutes, M (SD)	total	195 (110)	178 (95)	159 (87)	180 (117)
	NC	231 (122)	200 (98)	179 (87)	213 (125)
	NAC	159 (74)	160 (100)	149 (96)	140 (100)
	AC	137 (68)	139 (63)	118 (65)	137 (84)

Note. M = mean, SD = standard deviation; total = all patients, n = 99; NC = no chemotherapy, n = 55; AC = adjuvant chemotherapy, n = 23; NAC = neoadjuvant chemotherapy, n = 21.
